# High-Efficiency Metasurfaces with 2π Phase Control Based on Aperiodic Dielectric Nanoarrays

**DOI:** 10.3390/nano10020250

**Published:** 2020-01-31

**Authors:** Sihui Shang, Feng Tang, Xin Ye, Qingzhi Li, Hailiang Li, Jingjun Wu, Yiman Wu, Jun Chen, Zhihong Zhang, Yuanjie Yang, Wanguo Zheng

**Affiliations:** 1 School of Physics, University of Electronic Science and Technology of China, Chengdu 610054, China; 201721040131@std.uestc.edu.cn (S.S.); dr.yang2003@uestc.edu.cn (Y.Y.); 2 Research Center of Laser Fusion, China Academy of Engineering Physics, Mianyang 621900, Sichuan, China; tangfengf3@126.com (F.T.); yexin@caep.cn (X.Y.); dearlqz@163.com (Q.L.); jingjunwu163@163.com (J.W.); manmanna@yeah.net (Y.W.); chenjun19950110@163.com (J.C.); 3 Key Laboratory of Microelectronic Devices & Integrated Technology, Institute of Microelectronics of Chinese Academy of Sciences, Beijing 100029, China

**Keywords:** metasuface, Huygens, granting, metalens, phase control

## Abstract

In this study, the high-efficiency phase control Si metasurfaces are investigated based on aperiodic nanoarrays unlike widely-used period structures, the aperiodicity of which providing additional freedom to improve metasurfaces’ performance. Firstly, the phase control mechanism of Huygens nanoblocks is demonstrated, particularly the internal electromagnetic resonances and the manipulation of effective electrical/magnetic polarizabilities. Then, a group of high-transmission Si nanoblocks with 2π phase control is sought by sweeping the geometrical parameters. Finally, several metasurfaces, such as grating and parabolic lens, are numerically realized by the nanostructures with high efficiency. The conversion efficiency of the grating reaches 80%, and the focusing conversion efficiency of the metalens is 99.3%. The results show that the high-efficiency phase control metasurfaces can be realized based on aperiodic nanoarrays, i.e., additional design freedom.

## 1. Introduction

Due to the restricted permittivity and permeability, natural materials cannot manipulate electromagnetic waves arbitrarily and wilfully. Electromagnetic properties of metamaterials can be designed artificially, breaking the limit of natural materials and having great potentials in the applications of imaging [1,2], solar cells [3], holographic [4,5], etc. However, the disadvantages of high loss, fabrication challenges and strong dispersion [6] limit the possible applications of 3D metamaterial, resulting in the appearance of metasurfaces, i.e., sub-wavelength nanostructure (meta-atom) arrays. Metasurfaces can compress the thickness of traditional optical elements [7] to the sub-wavelength level, performing an arbitrary manipulation of amplitude [8,9,10,11], phase [12,13,14,15,16], and polarization [17,18,19]. Unlike plasmonic ones [20,21,22,23,24], dielectric metasurfaces [25] have made significant breakthroughs in optical refractive index sensors [26], biochemical sensing devices [27], and microwave sensors [28], particularly based on semiconductor materials like Si, have lower loss and higher efficiency [29], and compatibility with the CMOS facility.

Electromagnetic fields can excite both electric and magnetic dipoles in dielectric nanostructures [29,30]. It is shown that the simultaneous manipulation of electric and magnetic dipoles can achieve 2π phase control in a single-layer dielectric nanoarray. This kind of metasurface has a theoretical transmittance approaching 100%, i.e., Huygens source [29,31,32,33]. Recently, some reports have demonstrated that Huygens metasurface [29,31,34], using Si and TiO_2_ [35] as low-loss semiconductor materials, can realize high efficiency in 2π phase wavefront control. Metamaterials are expected to integrate with on-chip nanophotonic devices, and a variety of functional materials incorporated to reconfigurable or tunable metasurfaces is also presented [36,37]. Moreover, many studies have shown that MEMS tunable dielectric metasurfaces lenses pave the way for MEMS-integrated metasurfaces, which can be fully used as a platform for tunable and reconfigurable optical [38,39]. Existing studies on metasurfaces are mainly based on periodic nanoarrays of meta-atoms [14,17,30], of which the optical properties will deviate from the designed values when constructing metasurfaces, finally causing the undesired optical performance and low efficiency of metasurfaces.

The 830 nm wavelength laser is widely used in clinical and medical applications [40,41,42]. In this study, a method of constructing Huygens metasurfaces based on aperiodic Si nanoarrays is proposed to approach phase manipulation of 830 nm light, in which the edge to edge distance keeps constant. This approach can provide additional design freedom and improve metasurfaces’ performance. The studied metasurfaces can replace traditional gratings and lenses to conduct optical deflection, focusing, imaging, etc. Firstly, the optical properties of Si meta-atoms are studied by finite-difference time-domain (FDTD) simulations and the internal phase control mechanism is demonstrated based on the coupling of electric/magnetic dipoles. Then, a group of Si meta-atoms with 2π phase control is sought by sweeping the geometrical parameters. Finally, two example metasurfaces of grating and parabolic lens are constructed and analyzed numerically in the near-infrared range. The results show that the high-efficiency phase control metasurfaces can be realized based on aperiodic nanoarrays.

## 2. Design of Meta-atoms and Methods

All simulations in this study were implemented using FDTD Solutions, a commercial software package provided by Lumerical Solutions, Inc. The simulation region of meta-atoms was surrounded with periodic boundaries in the x-axis and y-axis directions and perfectly matched layers in the z-axis direction. The size of the nanoblocks was set from 180nm to 430nm. The height of nanoblocks was set at 132nm. The size effect on transmittance and phase was calculated. All objects, sources (S parameter), and monitors were laid in this simulation region. The PML boundary was used to the x-axis, y-axis, and z-axis for simulating optical devices. The TFSF (total-field scattered-field) source, all objects, and monitors were limited in the simulation region. The refractive indexes of silicon (Si) and glass (SiO_2_) come directly from the database of the FDTD software. 

**Figure 1:**
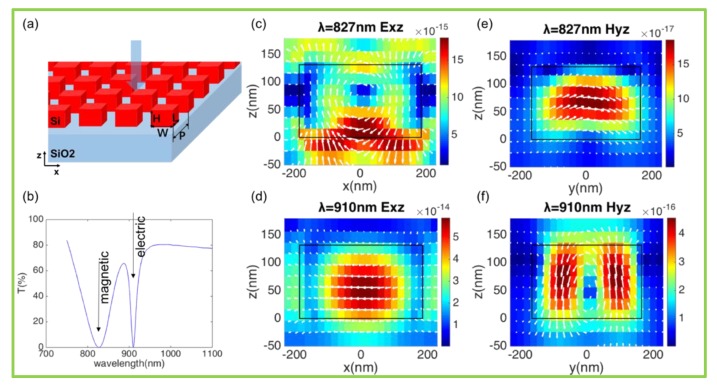
Schematic of meta-atoms and internal electromagnetic excitation. (**a**) Three-dimensional view of meta-atoms, i.e., a nanoblock array on top of the silica substrate. Nanoblocks’ height H = 132 nm, width W = 370 nm, length L = 330 nm, and period P = 460 nm. (**b**) Transmittance spectrum of meta-atoms with two dips for magnetic (*λ_m_* = 827 nm) and electric (*λ_e_* = 910 nm) resonances. (**c**,**d**) Electric/magnetic field distribution of the magnetic dipole, which is indicated by the vortex-like electric pattern, on two orthogonal cross-sections of the nanoblocks. (**e**,**f**) Electric/magnetic field distribution of the electric dipole, which is indicated by the vortex-like magnetic pattern, on two orthogonal cross-sections of the nanoblocks. Electric and magnetic field amplitudes are normalized to their maximum values in the study.

According to the analytical model of Huygens meta-atoms, the transmittance and reflection coefficients [29] can be expressed as below:

(1) $$t=1+\frac{2i\cdot \gamma_{e}\omega }{\omega_{e}^{2}-\omega^{2}-2i\gamma_{e}\omega }+\frac{2i\cdot \gamma_{m}\omega }{\omega_{m}^{2}-\omega^{2}-2i\gamma_{m}\omega }$$

(2) $$r=\frac{2i\cdot \gamma_{e}\omega }{\omega_{e}^{2}-\omega^{2}-2i\gamma_{e}\omega }-\frac{2i\cdot \gamma_{m}\omega }{\omega_{m}^{2}-\omega^{2}-2i\gamma_{m}\omega }$$

where $\omega_{e}$ and $\omega_{m}$ represent the resonant frequencies of the electric dipole and the magnetic dipole, respectively. $\gamma_{e}$ and $\gamma_{m}$ represents the damping coefficient of the electric/magnetic dipole. When $\omega =\omega_{e}=\omega_{m}$ and r=0, the individual non-reflective nanoblock can be regarded as an ideal Huygens source. Metasurfaces are composed of nanoblocks (i.e., meta-atom) with different sizes, arrangements, etc. Thus, the array of meta-atoms with the same parameters is investigated firstly, as shown in Figure 1. An array of nanoblocks, with height H = 132 nm, width W = 370 nm, length L = 330 nm, and the period P = 460 nm, is on top of silica substrate. The incidence is assumed to be a plane wave, propagating along the z-axis and being polarized along the x-axis. For light propagating through the array, the transmittance spectrum has two dips caused by the magnetic (λ_m_ = 827 nm) and electric (λ_e_ = 910 nm) resonances. The corresponding electromagnetic distributions are presented in Figure 1c–f, showing the patterns of magnetic/electric dipoles. When the two dipoles overlap via changing the nanoblocks’ geometrical parameters, 100% transmission can be realized theoretically. 

**Figure 2:**
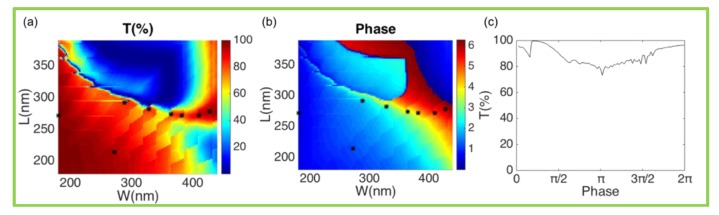
Design of meta-atoms. (**a**) Transmittance for nanoblocks on silica substrate as function of nanoblocks’ width W and length L with H = 132 nm and P = max(W,L) + 90 nm. (**b**) Corresponding phase variation of transmitted light through nanoblocks. (**c**) The highest transmittance achieved by the nanoblocks in the 0–2π full-phase space. The period P keeps constant max(W,L) + 90 nm. The black star markers indicate the eight meta-atoms selected to construct metasurfaces.

By tuning $\omega_{e}$, $\omega_{m}$, $\gamma_{e}$ and $\gamma_{m}$ via blocks’ sizes, *r* can be 0, i.e., the magnetic/electric dipoles overlap and the impedance matches with the surrounding medium. In the case, the transmission coefficient t is 100%, where a non-reflective Huygens source appears. Tuning the nanoblocks in the vicinity of the parameters at the match point, the phase of t can cover the 0-2π range and the transmittance is still near 100%. Transmittance and phase variation of light are calculated as a function of nanoblocks’ width *W* and length *L* with high H and period P are parameters that need to be optimized, as shown in Figure 2. To avoid the deviation of optical properties in constructing metasurfaces, the edge to edge distance *D* is kept constant when tuning the nanoblocks. The highest transmittance achieved by the nanoblocks in the 0–2π full-phase space is shown in Figure 2c. Eight points marked by stars are selected to construct metasurfaces. The transmittance and phase of the selected meta-atoms with size errors are shown in Figure A2.

The optical properties of meta-atoms are controlled by manipulating the charge oscillation with nanoboundaries. The effective optical properties are different from the bulk. The effective electrode rate $\alpha_{e}^{eff}$ and the effective magnetic polarizability $\alpha_{m}^{eff}$ are respectively expressed [31] as:

(3) $$j\omega \alpha_{e}^{eff}=\frac{2*\left(1-t-r\right)}{\sqrt{\mu /\varepsilon }\left(1+t+r\right)}$$

(4) $$j\omega \alpha_{m}^{eff}=\frac{2*\sqrt{\mu /\varepsilon }\left(1-t+r\right)}{\left(1+t-r\right)}$$

Here, ω is an angular frequency, ε and μ represent a dielectric constant and magnetic permeability in a vacuum, and *t* and *r* are transmission and reflection coefficients, respectively.

**Figure 3:**
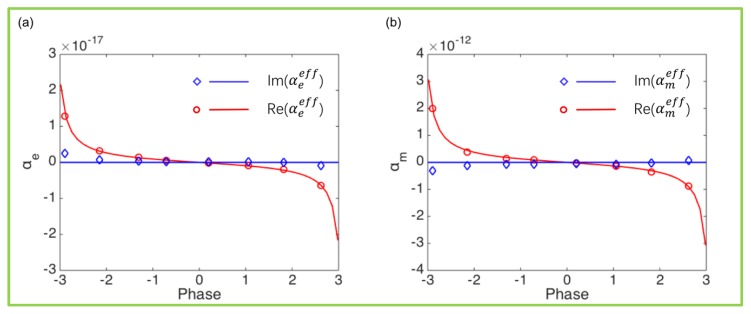
Effective (**a**) electric and (**b**) magnetic polarizabilities $\alpha_{e}^{\mathrm{eff}}/\alpha_{m}^{\mathrm{eff}}$ of the selected eight meta-atoms. The solid line is the ideal electromagnetic polarizabilities of Huygens meta-atoms.

The effective electric/magnetic polarizability of the selected eight meta-atoms in Figure 2 are shown in Figure 3. The solid red lines indicate the real part of the ideal effective polarizability when the transmittance is 100% while the blue one is the imaginary part. The red circles indicate the real part of the actual effective polarizability of the selected meta-atoms while the blue diamonds are the imaginary part. Moreover, we find that the real part of the meta-atomic polarizability designed in this paper is basically consistent with the ideal values, and there is a small amount of deviation in the imaginary part, which may be related to the absorption inside the meta-atoms. We calculated our Q factors and dephasing times of all the electromagnetic resonances of the Si nanoblocks, as shown in Figure A3.

## 3. Metasurface Construction and Simulation

**Figure 4:**
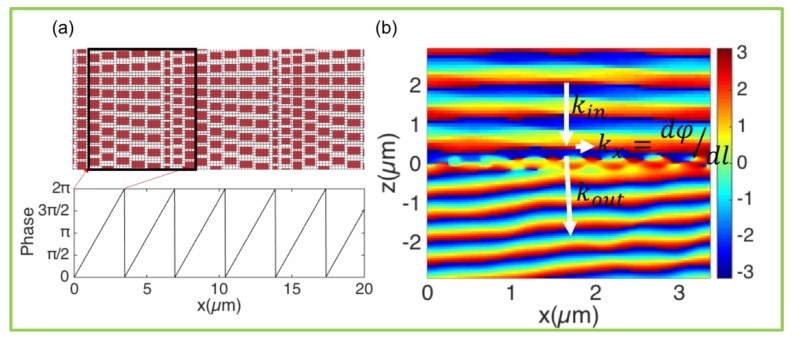
Metasurfaces of grating. (**a**) Top: schematics of grating composed of the eight meta-atoms in Figure 3, having the digital phase shift with π/4 increments covering the 2π range. Bottom: phase distribution function of the metasurface. (**b**) Phase distribution when a plane wave passes through the metasurface, showing deflection with angle 9.5°.

**Figure A1:**
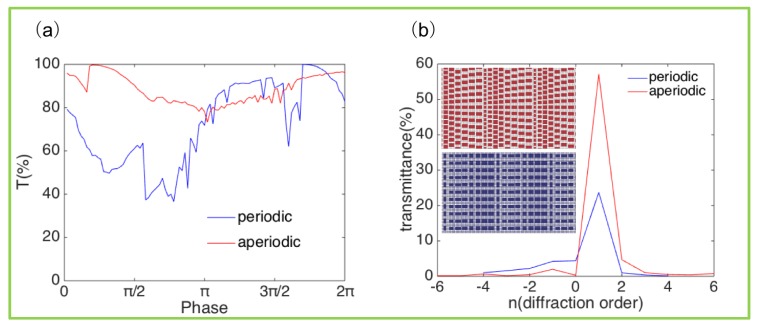
(**a**)The highest transmittance achieved by the comparison of periodic (blue) and aperiodic (red) nanoblocks in the 0–2π full-phase space, which shows that aperiodic significantly improves the corresponding efficiency. (**b**) Contrast diagram of far-field radiation intensity. The inserted red image is the grating composed of aperiodic meta-atoms, and the blue image is the grating composed of periodic meta-atoms.

**Figure A2:**
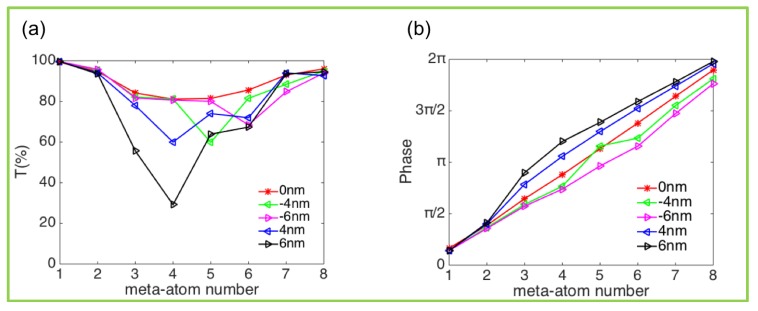
Transmittance and phase of the selected meta-atoms with size errors.

**Figure A3:**
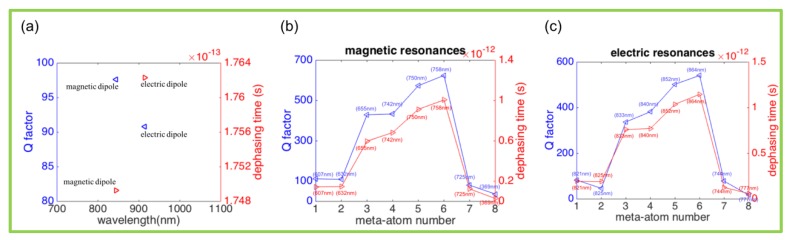
Q factors (blue marks) and dephasing times (red marks) of: (**a**) the electric dipole and magnetic dipole in Figure 1; (**b**) the magnetic resonances; and (**c**) electric resonances of the eight selected meta-atoms that are marked by stars in Figure 2.

Since the phase distribution function of the metasurface can be independently controlled by every single nanostructure in each microscopic region, independent phase manipulation can be performed on each point constituting the wavefront of the target beam. Therefore, we can design arbitrary phase functions based on the selected meta-atoms. We built a beam deflector (Figure 4) and simulated its performance. The phase function of abnormal refraction is $\varphi \left(x\right)=k_{x}\times x$, where $k_{x}$ is the phase gradient of the metasurface. The simulation results are shown in Figure 4b, which is the phase distribution on the x-z plane. The incident plane light is deflected. The theoretical angle of deflection $\theta =\arcsin\left[\left(d\varphi /dl\right)/\left(2\pi n/\lambda \right)\right]\approx 9.5^{{}^{\circ}}$ (where, λ is the wavelength of light in free space, and dφ/dl is the phase gradient in the x-direction), which is consistent with the simulation results. The transmitted light conversion efficiency of the sample reaches 80%. The transmission comparison between non-periodic and periodic arrays of meta-atoms is shown in Figure A1.

**Figure 5:**
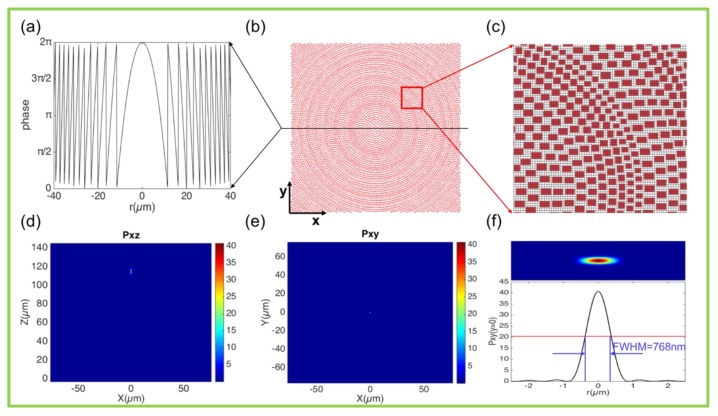
Metasurfaces of parabolic lens. (**a**) Phase-distribution function of the metalens in radial direction. (**b**) Top view of metalens composed of the eight meta-atoms in Figure 3, introducing the digital phase shift with π/4 increments covering the 2π range. (**c**) Partial enlarged details of the metasurface, which is marked by the red box in (b). (**d**,**e**) Power distributions of the focusing spot in the x–z (y = 0 nm) and x–y (z = 115.1 µm) planes, respectively. (**f**) Full width at half maximum (FWHM) of the focusing spot.

Furthermore, according to the phase function of the two-dimensional lens:

(5) $$\varphi \left(r,\lambda \right)=-\frac{2\pi }{\lambda }\left(\sqrt{r^{2}+f^{2}}-f\right)+\varphi_{0}\left(\lambda \right)$$

where f is the focal length, λ is the wavelength, and $\varphi_{0}\left(\lambda \right)$ is an arbitrary phase. The corresponding phase distribution is shown in Figure 5a. Based on the selected meta-atoms, a plane lens was constructed with focal length f = 120 µm, aperture D = 150 µm, as in Figure 5b,c. The plane light is focused at the focal plane position, as shown in Figure 5d,e. Figure 5d shows the power distribution of the focusing spot on the x-z plane, and the focal spot appears at the position of z = 115.1 µm. Figure 5e shows the power distribution of the focusing spot in the x-y plane, i.e., the focal plane. The upper part of Figure 5f is the x-y plane power distribution. The lower part is the power distribution with y = 0, and the half peak full width (FWHM, 768 nm) of the focal spot reaches the sub-wavelength magnitude. Compared to the metasurface based on periodic meta-atoms, our structure effectively avoids diffraction and interference. Furthermore, due to the sub-wavelength scale, the scattering of light is also reduced. Therefore, it achieved a focusing conversion efficiency (the power in the focused light spot (with diameter 3 × FWHM divided by the total power on the focal plane) of 99.3% for all the transmitted light. It is worth noting that the focal plane position is at z = 115.1 µm, which is slightly different from the designed focal length (120 µm), which may be related to the position of the main plane of the lens. On the other hand, it may also be related to the height of the meta-atoms.

**Figure 6:**
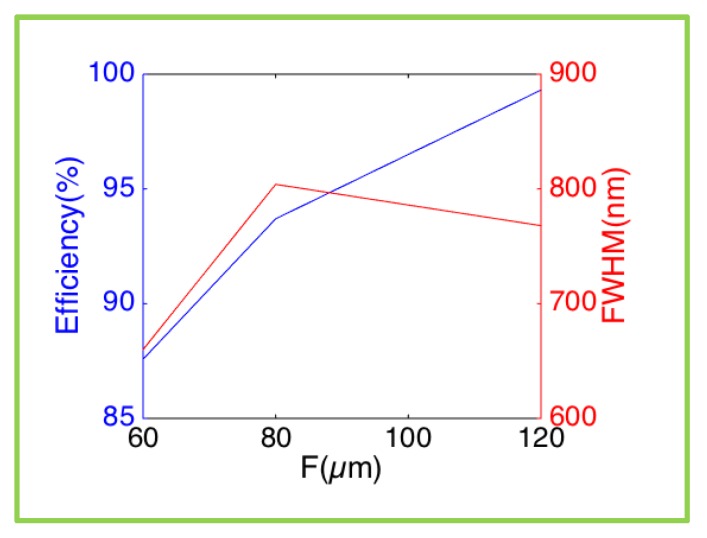
Focusing efficiency and FWHM of focus spot as function of the meta-less’ focal length f.

When changing the focus length of the meta-lens, i.e., f = 60 µm (D = 100 µm), f = 80 µm (D = 100 µm), and f = 120 µm (D = 150 µm), the corresponding focusing efficiency increases, as shown in Figure 6. A larger focal length f means a smaller phase gradient of the meta-lens, and therefore the phase function digitalization of lens has higher accuracy spatially based on the eight selected meta-atoms. The F-numbers of the three meta-lens are 0.6, 0.8, and 0.8, respectively. Therefore, the latter two lenses have similar FWHM values, 804 nm and 768 nm, bigger than the first one at 660 nm. Moreover, all the FWHM values are smaller than the wavelength of 830 nm.

## 4. Conclusions

To sum up, based on aperiodic Si nanoarrays, metasurfaces with 2π phase control are investigated. An abnormal refraction device with a deflection angle $9.5^{{}^{\circ}}$ is realized, and the conversion efficiency of transmitted light is up to 80%. Based on the phase function of the plane lens, a two-dimensional meta-lens is designed to achieve the perfect focusing with a focal length of 115.1 μm, an F-number of 0.8, a focal spot size of 768 nm, and the focusing conversion efficiency of the transmitted light reaches 99.3%. Most notably, we use aperiodic meta-atomic structures that greatly reduce the diffraction and interference effects of the source at the focal plane. Similarly, we can design corresponding optical metasurface devices according to any phase function. By combining with the current mature CMOS processing technology, large-scale industrial production of metasurface devices can be realized at a low cost, which can be widely used in industrial, military, and other fields.

## Appendix A

The transmission comparison between non-periodic and periodic arrays of meta-atoms is shown in Figure A1. We can design more parameters without being limited by periodicity. As shown Figure A1a, compared with the periodic meta-atoms, the transmittance of aperiodic structures is higher. Moreover, for the far-field radiation intensity of the grating, compared with the periodic meta-atoms, the first-order diffraction order efficiency of the metasurfaces composed of aperiodic meta-atoms is higher, and the efficiency of the other orders is lower, which shown the advantage of the aperiodic meta-atoms, as shown Figure A1b.

The transmittance and phase of the selected meta-atoms with size errors are shown in Figure A2. The red curves with stars show the ideal case in which the selected meta-atoms have the sizes in Figure 2. If the fabrication has an error of ±4 nm, the transmittance and phase are shown by the blue and green curves. The transmittance of meta-atoms numbered 1, 2, 7, and 8 stays almost the same while the others show a drop of 20%. When the error increases to −6 nm, the transmittance decreases to 25%. However, the error of +6 nm introduces the transmittance change is similar to the error case of ±4 nm. In terms of phase, a bigger error brings a bigger offset from the desirable phase. The positive error makes the phase bigger than the desired value while the negative error brings a phase smaller than the desired value.

We calculated our Q factors and dephasing times [43,44] of all the electromagnetic resonances of the Si nanoblocks. As shown in Figure A3a, the Q factors and dephasing time of the electromagnetic resonances of the Si nanoblock in Figure 1 are calculated. Figure A3b,c shows the Q factors and dephasing time of the electromagnetic resonances of the eight selected meta-atoms in Figure 2.

## References

[1] Lin R.J., Su V.C., Wang S., Chen M.K., Chung T.L., Chen Y.H., Kuo H.Y., Chen J.W., Chen J., Huang Y.T. (2019). Achromatic Metalens Array for Full-Colour Light-Field Imaging. Nat. Nanotechnol..

[2] Khorasaninejad M., Chen W.T., Devlin R.C., Oh J., Zhu A.Y., Capasso F. (2016). Metalenses at Visible Wavelengths: Diffraction-Limited Focusing and Subwavelength Resolution Imaging. Science.

[3] Pillai S., Catchpole K.R., Trupke T., Green M.A. (2007). Surface Plasmon Enhanced Silicon Solar Cells. Br. J. Appl. Phys..

[4] Deng Z.L., Zhang S., Wang G.P. (2016). A Facile Grating Approach Towards Broadband, Wide-Angle and High-Efficiency Holographic Metasurfaces. Nanoscale.

[5] Liu D., Wu J., Guo S. (2018). The Generation of Three-Dimensional Curved Beams Based on Holographic Metasurface. Opt. Express.

[6] Zhang Z.H., Shang S.H., Li M.N., Wu S.Y., Zhu Q.S., Wu M.H., Teng B.H. (2018). Fdtd Simulation: Refractive Index and Single-Object Sensing Using a Whispering-Gallery-Modes Microring Resonator. J. Lightw. Technol..

[7] Yang Y., Zhao Q., Liu L., Liu Y., Rosales-Guzmán C., Qiu C.W. (2019). Manipulation of Orbital-Angular-Momentum Spectrum Using Pinhole Plates. Phys. Rev. Appl..

[8] Liu L., Zhang X., Kenney M., Su X., Xu N., Ouyang C., Shi Y., Han J., Zhang W., Zhang S. (2014). Broadband Metasurfaces with Simultaneous Control of Phase and Amplitude. Adv. Mat..

[9] Shao T., Sun L., Li W., Zhou X., Wang F., Huang J., Ye X., Yang L., Zheng W. (2019). Understanding the Role of Fluorine-Containing Plasma on Optical Properties of Fused Silica Optics During the Combined Process of Rie and Dce. Opt. Express.

[10] Sun L., Liu H., Huang J., Ye X., Xia H., Li Q., Jiang X., Wu W., Yang L., Zheng W. (2016). Reaction Ion Etching Process for Improving Laser Damage Resistance of Fused Silica Optical Surface. Opt. Express.

[11] Luo J., Yang Y., Yao Z., Lu W., Hou B., Hang Z.H., Chan C.T., Lai Y. (2016). Ultratransparent Media and Transformation Optics with Shifted Spatial Dispersions. Phys. Rev. Lett..

[12] Zhu B.O., Zhao J., Feng Y. (2013). Active Impedance Metasurface with Full 360° Reflection Phase Tuning. Sci. Rep..

[13] Arbabi A., Horie Y., Bagheri M., Faraon A. (2015). Dielectric Metasurfaces for Complete Control of Phase and Polarization with Subwavelength Spatial Resolution and High Transmission. Nat. Nanotechnol..

[14] Yu Y.F., Zhu A.Y., Paniagua-Domínguez R., Fu Y.H., Luk’yanchuk B., Kuznetsov A.I. (2015). High-Transmission Dielectric Metasurface with 2π Phase Control at Visible Wavelengths. Laser & Photonics Rev..

[15] Pun E.Y.B., Wong P.W.H., Li G., Chen S., Pholchai N., Reineke B., Cheah K.W., Zentgraf T., Zhang S. Nonlinear Metasurface for Continuous Phase Control. Proceedings of the 2016 IEEE MTT-S International Conference on Numerical Electromagnetic and Multiphysics Modeling and Optimization (NEMO).

[16] Wang W., Guo C., Tang J., Zhao Z., Wang J., Sun J., Shen F., Guo K., Guo Z. (2019). High-Efficiency and Broadband near-Infrared Bi-Functional Metasurface Based on Rotary Different-Size Silicon Nanobricks. Nanomaterials.

[17] Yang Y., Wang W., Moitra P., Kravchenko I.I., Briggs D.P., Valentine J. (2014). Dielectric Meta-Reflectarray for Broadband Linear Polarization Conversion and Optical Vortex Generation. Nano Lett..

[18] Chen W.T., Yang K.Y., Wang C.M., Huang Y.W., Sun G., Chiang I.D., Liao C.Y., Hsu W.L., Lin H.T., Sun S. (2014). High-Efficiency Broadband Meta-Hologram with Polarization-Controlled Dual Images. Nano Lett..

[19] Shibanuma T., Maier S.A., Albella P. (2018). Polarization Control of High Transmission/Reflection Switching by All-Dielectric Metasurfaces. Appl. Phys. Lett..

[20] Huang Y.W., Chen W.T., Tsai W.Y., Wu P.C., Wang C.M., Sun G., Tsai D.P. (2015). Aluminum Plasmonic Multicolor Meta-Hologram. Nano Lett..

[21] Pors A., Nielsen M.G., Eriksen R.L., Bozhevolnyi S.I. (2013). Bozhevolnyi. Broadband Focusing Flat Mirrors Based on Plasmonic Gradient Metasurfaces. Nano Lett..

[22] Peyskens F., Subramanian A.Z., Neutens P., Dhakal A., Van Dorpe P., Le Thomas N., Baets R. (2015). Bright and Dark Plasmon Resonances of Nanoplasmonic Antennas Evanescently Coupled with a Silicon Nitride Waveguide. Opt. Express.

[23] Wang W., Zhao Z., Guo C., Guo K., Guo Z. (2019). Spin-Selected Dual-Wavelength Plasmonic Metalenses. Nanomaterials.

[24] Miscuglio M., Borys N.J., Spirito D., Martín-García B., Zaccaria R.P., Weber-Bargioni A., Schuck P.J., Krahne R. (2019). Planar Aperiodic Arrays as Metasurfaces for Optical near-Field Patterning. ACS Nano.

[25] Chen S., Chen Z., Liu J., Cheng J., Zhou Y., Xiao L., Chen K. (2019). Ultra-Narrow Band Mid-Infrared Perfect Absorber Based on Hybrid Dielectric Metasurface. Nanomaterials.

[26] Zhang J., Liu W., Zhu Z., Yuan X., Qin S. (2014). Strong Field Enhancement and Light-Matter Interactions with All-Dielectric Metamaterials Based on Split Bar Resonators. Opt. Express.

[27] Ahmadivand A., Karabiyik M., Pala N. (2015). Inducing Multiple Fano Resonant Modes in Split Concentric Nanoring Resonator Dimers for Ultraprecise Sensing. J. Opt..

[28] Semouchkina E., Duan R., Semouchkin G., Pandey R. (2015). Sensing Based on Fano-Type Resonance Response of All-Dielectric Metamaterials. Sensors.

[29] Decker M., Staude I., Falkner M., Dominguez J., Neshev D.N., Brener I., Pertsch T., Kivshar Y.S. (2015). High-Efficiency Dielectric Huygens’ Surfaces. Adv. Opt. Mat..

[30] Shalaev M.I., Sun J., Tsukernik A., Pandey A., Nikolskiy K., Litchinitser N.M. (2015). High-Efficiency All-Dielectric Metasurfaces for Ultracompact Beam Manipulation in Transmission Mode. Nano Lett..

[31] Zhao W., Jiang H., Liu B., Song J., Jiang Y., Tang C., Li J. (2016). Dielectric Huygens’ Metasurface for High-Efficiency Hologram Operating in Transmission Mode. Sci. Rep..

[32] Chen M., Kim M., Wong A.M., Eleftheriades G.V. (2018). Eleftheriades. Huygens’ Metasurfaces from Microwaves to Optics a Review. Nanophotonics.

[33] Kim M., Wong A.M., Eleftheriades G.V. (2014). Optical Huygens’ Metasurfaces with Independent Control of the Magnitude and Phase of the Local Reflection Coefficients. Phys. Rev. X.

[34] Sun Z., Sima B., Zhao J., Feng Y. (2019). Electromagnetic Polarization Conversion Based on Huygens’ Metasurfaces with Coupled Electric and Magnetic Resonances. Opt. Express.

[35] Li X., Chen G., Yang L., Jin Z., Liu J. (2010). Multifunctional Au-Coated Tio2 Nanotube Arrays as Recyclable Sers Substrates for Multifold Organic Pollutants Detection. Adv. Funct. Mater..

[36] Hsiao H.H., Chu C.H., Tsai D.P. (2017). Fundamentals and Applications of Metasurfaces. Small Methods.

[37] Arbabi A., Briggs R.M., Horie Y., Bagheri M., Faraon A. (2015). Efficient Dielectric Metasurface Collimating Lenses for Mid-Infrared Quantum Cascade Lasers. Opt. Express.

[38] Arbabi E., Arbabi A., Kamali S.M., Horie Y., Faraji-Dana M., Faraon A. (2018). Mems-Tunable Dielectric Metasurface Lens. Nat. Commun..

[39] Colburn S., Zhan A., Majumdar A. (2017). Tunable Metasurfaces Via Subwavelength Phase Shifters with Uniform Amplitude. Sci. Rep..

[40] Stadler I., Lanzafame R.J., Evans R., Narayan V., Dailey B., Buehner N., Naim J.O. (2001). 830-Nm Irradiation Increases the Wound Tensile Strength in a Diabetic Murine Model. Lasers Surg. Med. Off. J. Am. Soc. Laser Med. Surg..

[41] Hagiwara S., Iwasaka H., Okuda K., Noguchi T. (2007). Gaalas (830 Nm) Low-Level Laser Enhances Peripheral Endogenous Opioid Analgesia in Rats. Lasers Surg. Med..

[42] Chow R.T., Heller G.Z., Barnsley L. (2006). The Effect of 300 Mw, 830 Nm Laser on Chronic Neck Pain: A Double-Blind, Randomized, Placebo-Controlled Study. Pain.

[43] Ahmadivand A., Gerislioglu B., Ramezani Z. (2019). Gated Graphene Island-Enabled Tunable Charge Transfer Plasmon Terahertz Metamodulator. Nanoscale.

[44] Sönnichsen C., Franzl T., Wilk T., Von Plessen G., Feldmann J. (2002). Plasmon Resonances in Large Noble-Metal Clusters. New J. Phys..

